# Toward a hemorrhagic trauma severity score: fusing five physiological biomarkers

**DOI:** 10.1186/s12967-020-02516-4

**Published:** 2020-09-14

**Authors:** Ankita Bhat, Daria Podstawczyk, Brandon K. Walther, John R. Aggas, David Machado-Aranda, Kevin R. Ward, Anthony Guiseppi-Elie

**Affiliations:** 1 Center for Bioelectronics, Biosensors and Biochips (C3B®), Department of Biomedical Engineering, Texas A&M University, College Station, TX 77843 USA; 2 Department of Process Engineering and Technology of Polymer and Carbon Materials, Wroclaw University of Science and Technology, Norwida 4/6, 50-373 Wroclaw, Poland; 3 Department of Cardiovascular Sciences, Houston Methodist Institute for Academic Medicine and Houston Methodist Research Institute, 6670 Bertner Ave, Houston, TX 77030 USA; 4 Departments of Emergency Medicine and Biomedical Engineering, Michigan Center for Integrative Research in Critical Care, University of Michigan, Ann Arbor, MI 48109 USA; 5 Department of Surgery, Division of Acute Care Surgery, University of Michigan, Ann Arbor, MI 48109 USA; 6 Department of Electrical and Computer Engineering, Texas A&M University, College Station, TX 77843 USA; 7 ABTECH Scientific, Inc, Biotechnology Research Park, 800 East Leigh Street, Richmond, VA 23219 USA

**Keywords:** Decision-making, Hemorrhage, Trauma care, DATA fusion, Risk stratification, Triage

## Abstract

**Background:**

To introduce the Hemorrhage Intensive Severity and Survivability (HISS) score, based on the fusion of multi-biomarker data; glucose, lactate, pH, potassium, and oxygen tension, to serve as a patient-specific attribute in hemorrhagic trauma.

**Materials and methods:**

One hundred instances of Sensible Fictitious Rationalized Patient (SFRP) data were synthetically generated and the HISS score assigned by five clinically active physician experts (100 [5]). The HISS score stratifies the criticality of the trauma patient as; low(0), guarded(1), elevated(2), high(3) and severe(4). Standard classifier algorithms; linear support vector machine (SVM-L), multi-class ensemble bagged decision tree (EBDT), artificial neural network with bayesian regularization (ANN:BR) and possibility rule-based using function approximation (PRBF) were evaluated for their potential to similarly classify and predict a HISS score.

**Results:**

SVM-L, EBDT, ANN:BR and PRBF generated score predictions with testing accuracies (majority vote) corresponding to 0.91 ± 0.06, 0.93 ± 0.04, 0.92 ± 0.07, and 0.92 ± 0.03, respectively, with no statistically significant difference (p > 0.05). Targeted accuracies of 0.99 and 0.999 could be achieved with SFRP data size and clinical expert scores of 147[7](0.99) and 154[9](0.999), respectively.

**Conclusions:**

The predictions of the data-driven model in conjunction with an adjunct multi-analyte biosensor intended for point-of-care continual monitoring of trauma patients, can aid in patient stratification and triage decision-making.

## Background

Trauma accounts for 47% of mortalities in individuals 1–46 years of age in the United States [1, 2]. Trauma-induced hemorrhage with its attendant peripheral vasoconstriction [3, 4] insulin resistance [5], hyperlactatemia, [6–8] acidosis [9], hyperkalemia [10, 11] and hypoxia can rapidly lead to death or may be followed by Multiple Organ Dysfunction Syndrome (MODS), a consequence of a “cytokine storm”, which can also be fatal [9, 12]. The field triage decision scheme for the national trauma triage protocol provides guidelines to identify the status of the patient [13]. The physiological criteria includes identification of vital signs such as; systolic blood pressure (Hypotension < 90 mmHg), [14–16], abnormal respiratory rate (< 10 or > 29 breaths per minute) [13], abnormal heart rate (Tachycardia > 100 beats per minute) [17], and the Glasgow coma scale (≤ 13) [18, 19]. The Glasgow coma scale categorizes the patients according to the severity of brain injury. Simple Triage and Rapid Treatment (START) is the commonly used algorithm for mass casualty triage in the USA [20–23], which is used in conjunction with secondary triage for Secondary Assessment of Victim Endpoint (SAVE) when the resource supply is restricted [20]. START and SAVE employ criteria such as respiratory rate, cognitive function (ability to listen and respond to commands), and radial pulse to identify the category for triage. Another example is the Injury Severity Score (ISS) [24] based on the Abbreviated Injury Scale (AIS) system which aggregates the assessed injury to six regions of the body and establishes correlations with mortality and morbidity. American College of Surgeons Committee on Trauma (ACS COT) aims to improve care by supporting programs for injury prevention [25]. An additional data source is the MIMIC-III data set, a freely accessible critical care database attributable to Johnson et al. [26]. MIMIC-III represents global vital signs and physiological waveforms; it does not contain data for hemorrhaging patients consisting of molecular biomarkers such as glucose, lactate, potassium, pH and oxygen tension. A MODS severity score was developed by Marshall et al. [27], wherein a score (0–4) is applied following physiologic measurement of dysfunction in 6 organ systems (i) respiratory function (pO_2_/FIO_2_ ratio), (ii) renal function (serum creatinine), (iii) liver function (serum bilirubin), (iv) cardiovascular function (PAR), (v) Hematologic (Platelet count) and (vi) Neurologic (Glasgow Coma Score). The total number of input points were then added to achieve a score corresponding to the patient’s ICU mortality %, hospital mortality %, and ICU stay.

Since the introduction of the MODS score, new rapidly deployable micro-analytical technologies have enabled measurement of key physiological indicators and the opportunity for the emergence of scores based on molecular biomarkers of physiological stress. A Hemorrhage Severity and Survivability Score (HISS) is herein introduced to allow for patient stratification. This stratification is made possible by the fusion of micro-analytical measurements of multiple physiological biomarkers [28]. HISS is a severity index intended as an adjunct to inform healthcare providers about the criticality of traumatic hemorrhage. This information would assist them in the delivery of timely and appropriate attention and care. HISS, therefore, is proposed to help in timely triage and in the stabilization of the most critically ill patients, and as a consequence, reduce patient mortality.

An adjunct device in the form of an indwelling biosensor system, the Physiologic Status Monitoring (PSM) Biochip, has been proposed and is under active development to help healthcare providers of trauma care in mass military and civilian triage situations [29, 30]. A dual-responsive biosensor for glucose and lactate has been proposed, designed, fabricated and successfully tested in rodent and piglet animal models of hemorrhaging trauma [31]. The PSM Biochip is a bio-SONDE; an indwelling device which measures, monitors and wirelessly transmits physicochemical information from within a victim of hemorrhaging trauma [29]. The bio-SONDE capable of acquiring the relevant physiological data pertinent to hemorrhagic shock states is the potential source of the data for subsequent fusion. When implanted intramuscularly, the PSM- Biochip enables the continuous, real-time monitoring of the patient’s physiological status via the key biomarkers; glucose, lactate, pH, potassium and oxygen tension. Such a system has the potential to go beyond single immediate datum (stat) capability to reveal evolving and predicted temporal trend status. This bio-SONDE is combined with a wireless processing hardware and a software algorithm to enable data fusion from the five identified biomarker analytes. This system would potentially guide evidence-based decision-making [32] derived from the real-time pathophysiological profile of the patient.

The present work evaluates multiple data fusion algorithms and seeks to identify the minimum patient and expert data sets needed to arrive at accurate predictions. The goal is to arrive at reliable and confident patient stratification decisions using the HISS Score. The present focus is on molecular biomarkers of pathophysiology as supplements to the traditional gross indicators for the development of biomarker monitoring systems at this scale for disease states such as traumatic shock [33]. Molecular indicators such as changes in oxygen tension may be earlier indicators of physiological stress than global vital signs. There are clear biochemical interactions among the identified variables. For example, glucose and lactate are related via the Cory Cycle. Lactate and acidosis (pH) are directly related. Indeed, there are statistical interactions among these variables. For example, some variables are known to swing quite widely during hemorrhage, such as glucose and lactate. Other variables are early onset indicators while others are late onset indicators, such as acidosis and potassium.

The main contributions of this study are (i) the establishment of a novel HISS score, (ii) the generation and use of Sensible Fictitious Rationalized Patient (SFRP) data, (iii) the prediction of the size of the patient and expert data set needed to achieve 99.0 and 99.9% accuracy in the predictions of HISS scores, and (iv) recognition of the inter-expert variability and strong intra-expert consistency in expert scoring data. Here, multiple fictitious patient physiological status data are produced and multiple individual experts score the data. A key consideration is thus the real-time fusion of disparate pathophysiological data to yield an actionable HISS score. Such data integration has medical and biomedical engineering applications such as in rapid, wearable health monitoring and internet of things (IoT) monitoring [34–35]. Data fusion can also be applied to implantable devices to generate data telemetry systems [36] with patient profiles [37]. Decision trees [38], support vector machine, neural networks [39], uncertainty index [40] and hybrid intelligent systems consisting of fuzzy logic and genetic algorithms [41] have been employed as classification approaches for data fusion in medicine. Decision tree classifiers were used to build a classification model in the form of a tree from the patient biomarker data [42]. The classifier provides a score for the data by testing each attribute and sorting and classifying particular instances in the data [43]. Ensemble bagged decision trees helped to reduce variance by the ‘bagging’ effect [44]. The support vector machine classifier [45] makes use of an optimal hyperplane and calculates the margin or the distance of the points from the hyperplane [46]. The points closest to the hyperplane are called the support vectors [47]. Support vector machines are often used because they are robust [48] and fast [45]. Neural networks mimic the structure of biological neurons, have input, output and/or hidden layers, and propagate to adjust the weights between the elements of the networks [49]. They are often used because of their value in tuning of data [50]. Genetic algorithms are employed to find an optimal solution for systems based on natural evolution [51] and have been used in time-series based neural networks [52] and in steady-state gene regulatory networks [53]. Similar approaches for the application of artificial intelligence in medicine and for developing a score for patients in the ICU [54] include the DeepSOFA [54], an automated alert functions for the patient status [55]. Decision support-systems employing an artificial intelligence clinician for sepsis in the ICU have also been generated [56].

Expert scoring of pathophysiological data may be incomplete or uncertain due to fuzziness or imprecision, and in some cases may be erroneous. Possibility theory [57] is a framework that is particularly applicable to expert knowledge. Unlike probability theory, possibility theory uses a pair of dual set-functions, namely possibility and necessity measures which make it capable of representing partial ignorance [58]. The possibility rule-based classification using function approximation (PRBF) algorithm has been shown to successfully handle the uncertainty in class labels of data and make an efficient use of the available data provided in the incomplete expert evaluation, a condition which is generally neglected in traditional supervised learning techniques [59]. Possibility labels may be directly extracted from an expert [60] by (1) the expert weighting the possibility of data belonging to each of the given c classes by a number between 0 and 1, or (2) to use possibility histograms from an empirical distribution of multiple expert opinions.

In the absence of actual viable penta-analyte patient data, synthetic data must be developed. Thus, a secondary objective of this work was to develop a synthetic data generation algorithm that produces Sensible Fictitious Rationalized Patient (SFRP) data. The SFRP algorithm creates a hidden seed layer and then generates biomarker values with filters to add noise/fuzziness and introduce variance to the five physiological biomarkers of interest. Practitioner input was sought in refining the filters and noise/fuzziness for each biomarker. The five biomarker values for each SFRP maps to a single output, the HISS score. The SFRP data were then shared with practicing physician experts who provided their individually rationalized HISS scores. Thus, the physicians’ scores serve as the ground truth but carry the inherent uncertainty born from disagreement among experts. Multiple SFRP data sets scored by a single expert, allowed an assessment of intra-professional variance. Correspondingly, multiple physicians providing ground truths of a single SFRP data set allowed accommodation of inter-professional variations. Multiple physician experts, given the results of a single set of measurements of physiological biomarkers, evaluate the status of patients in the form of a HISS score. In the decision-making processes, which incorporates bioanalytical diagnostic data and expertly sourced scores, uncertainty is inevitable. That is, given a reported set of measurements of the five biomarkers for a patient, different physicians may provide different evaluations, i.e. different scores, to represent the status of the patient. In such cases, it is possible to represent the uncertain scores in the form of a range of values. The generated data were used to make predictions for the status of the hemorrhaging patients by training a decision tree classifier and rule-based evolutionary classifier [58] to handle uncertainty in scores. The results of training models are presented in terms of their prediction accuracies. Furthermore, this allowed forecasting of the size of the patient data set and the number of clinician experts required to achieve stratification accuracies of 99% and 99.9%.

## Materials and methods

On-line data engines were searched for the availability of anonymized actual patient biomarker data for the hemorrhaging trauma patient (glucose, lactate, pH, potassium and oxygen tension). Public databases are available with specific datasets such as vital signs but they did not contain sufficient biomolecular data elements for the traumatic hemorrhage. Owing to necessary HIPAA-based security policies at hospitals, actual data for hemorrhaging patients could not be directly accessed. Access to diagnostic data sets under appropriate approvals is being pursued. Accordingly, the classification methods were each employed on the synthetically derived Sensible Fictitious Rationalized Patient (SFRP) data.

### Patient data generation-Sensible Fictitious Rationalized Patient data and evaluation by practitioners

In lieu of actual patient data, synthetic (SFRP) data sets were generated via a scripted algorithm in Python 3.7.0. The flowchart for the SFRP data set generator is based on the pathophysiological data in Table 1. The general algorithm begins with a seeded hidden layer of HISS scores that ranged from low(0) to severe(4). The initial seeding distribution for trauma scores was evenly distributed among the five levels. Each of the five biomarker values associated with each level was subsequently filled by randomly selecting a value from within a pathophysiological range that can be attributed to that trauma level (based on normal physiologic values and specific trauma and hemorrhage perturbations). The noise was introduced by controlling the relative level of deviation from initially seeded values into other trauma regimes—i.e. letting initially chosen values drift into other regimes not originally occupied by the primary, hidden trauma seed score. Glucose noise was based on potentially convoluting scenarios (adrenergic response) or by a simple, tunable probability of taking on a value, not within the seeded range. Lactate was similarly assigned. Potassium noise was added via post mathematical calculation. Acidosis (pH) noise was introduced by allowing for physiologically normal values to be taken at any hidden seed (with the rationale being that pH is a late and severe biomarker). Oxygen tension (pO_2_) noise was introduced via a convoluting scenario (respiratory compensation based on pH—determined randomly) and simple, random noise. The algorithm, in the most direct sense, allowed for initial seed values to bleed over into other regimes and create data that was confounded. For proof of concept, random number generators of no bias were used—although extension into Gaussian and other distributions may be readily implemented.

**Table 1:** Bounded pathophysiological ranges of key biomarkers of physiological stress in the hemorrhaging trauma patient

Pathophysiological r**ange**			
Analyte	Low	Normal	High
Glucose	*Hypoglycemia* *< 3.88* *mM* *< 70* *mg/dL*	Euglycemia 3.88–5.50 mM 70-99 mg/dL	Hyperglycemia >5.50–10.00 mM 99–180 mg/dL
Lactate	Hypolactatemia <0.50 mM	Eulactatemia 0.50–2.00 mM	*Hyperlactatemia* *> 2.00–4.00* *mM*
Potassium	Hypokalemia (< 3.50 mM)	Eukalemia 3.50-5.50 mM	*Hyperkalemia* *(> 5.50* *mM)*
pH	*Acidosis* *(< 7.35)*	7.35-7.45	Alkalosis (> 7.45)
pO_2_	*Hypoxia* *< 5.18* *mM* *< 100* *mmHg*	5.18-6.22 mM 100-120 mmHg	Hyperoxia (> 6.22 mM) >120 mmHg

Italicized entries relate to an example implementation of the SFRP data generator, explained further in the text

The algorithm may be run for any number of synthetic patients to generate SFRP data sets for each. For assessment, the initial hidden layer seeding was not output and was not revealed to any evaluator of the datasets. Potassium was determined via the empirical relationship from Burnell et al. [61], which details that a 0.1 unit drop in pH raises the [K^+^] by 0.6 mM. This is implemented in pseudocode in the following way for each output in (1):

1 $$\left[ {K^{ + } } \right]_{i} = random\left( {\left[ {K^{ + } } \right]_{normal} } \right) + \left( {7.35 {-} pH\left[ i \right]} \right)*6$$

where $\left[ {K^{ + } } \right]_{i}$ denotes the potassium of the $i$th patient, and $pH\left[ i \right]$ that patient’s pH level generated earlier. The random function action yields a normal potassium concentration within physiologic ranges, and is then altered if the pH of the patient is abnormal via the relation described. This concept is illustrated for the entries shown in italic in Table 1.

In this way a complete set of Sensible Fictitious Rationalized Patient data for n + 25 = 100 fictitious avatars (not patients) were created and ported into an excel spreadsheet for expert scoring and fusion considerations. Empirical relationships among the biomarker variables are possible and are being explored to enhance the robustness of the SFRP data sets. Table 2 shows a possible outcome for generating the training and testing data sets using the SFRP data generator. Accordingly, 100 unique Sensible Fictitious Rationalized Patient (SFRP) data sets were scored by five clinical experts. Each of the five experts assigned a HISS score, valued 0–4, to each penta-analyte data set while providing a rationale for their selection of the assigned score for a particular patient (0 = LOW, 1 = GUARDED, 2 = ELEVATED, 3 = HIGH, 4 = SEVERE). This resulted in a multi-class/expert framework [62] for the model-based predictions.

**Table 2:** Partial data set for “fictitious patients”, including training data set (1 to n) and testing data set (n + 1 to n + 25) generated using the Sensible Fictitious Rationalized Patient (SFRP) data generator and corresponding expert assigned Hemorrhage Intensive Severity and Survivability (HISS) score

Fictitious Patient	Sensible Fictitious Rationalized Patient (SFRP) Data					HISS				
	Glucose (mg/dl)	Lactate (mmol/l)	pH	Potassium (mmol/l)	pO_2_ (mmHg)	D1	D2	D3	D4	D5
1	70	2.7	7.42	5.10	78	1	1	1	1	0
2	160	6.0	7.11	6.14	44	4	2	3	3	3
*n*	41	9.7	7.26	4.84	97	3	3	4	3	3
..	..	..	..	..	..	..	..	..	..	..
*n *+ 1	123	3.3	7.41	5.00	86	UD	UD	UD	UD	UD
*n *+ 2	49	8.7	7.13	5.92	53	UD	UD	UD	UD	UD
..	..	..	..	..	..	..	..	..	..	..
*n *+ 25	220	8.6	7.23	4.52	92	UD	UD	UD	UD	UD

UD = undeclared i.e. assigned by the experts but predicted by the algorithms

### Classification algorithms

**Fig. 1:**
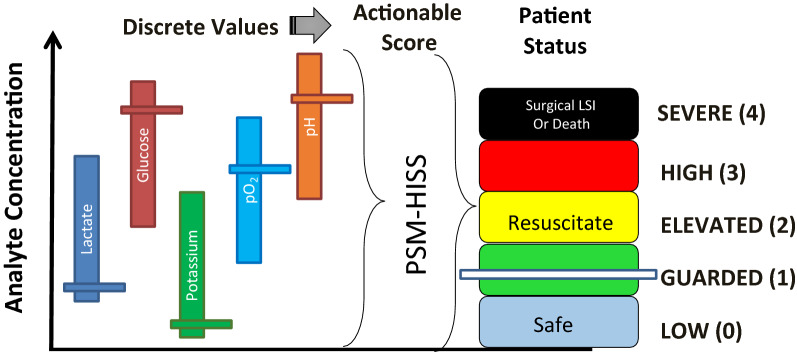
Immediate and continual measurement of key biomarkers may serve as a “gauge” for identifying shock states. Discrete values from the five indwelling biosensors are fused into a single actionable Hemorrhage Intensive Severity and Survivability (HISS) score [1]. This score is further stratified into five, color-coded levels, ‘SEVERE (4)’ being the most critical and ‘LOW (0)’ corresponding to expectant

Data from different sources can be fused via estimation, association and decision fusion [63]. Multi-class [64] linear support vector machines (SVM-L), ensemble bagged decision tree (EBDT), artificial neural network with Bayesian regularization algorithm (ANN:BR) and possibility rule-based using function approximation (PRBF) classifiers were used to classify the SFRP data sets. Figure 1 shows the concept for a fused score from the data of the five biomarkers. Five unique data sets, each of size 100, corresponding to the pathophysiological profile of 100 fictitious patients and along with the HISS scores of five healthcare provider experts: [100][D1], [100][D2], [100][D3] [100][D4] and [100][D5] were thus created from the available 100 penta-biomarker, patient data sets.

The multi-class ensemble bagged decision tree and linear support vector machine classifiers were used for predictions over the entire data sets (D1–D5). Neural networks [65] were used to determine the adequate number of training size for accurate predictions over the five data sets. Possibility rule-based classifiers were used to capture the uncertainty in the responses of the experts over the five data sets.

#### Multi-class linear support vector machine and ensemble bagged decision tree classifiers

For both support vector machine and decision tree classification models, a set of hyper-parameters was tuned and the model with the highest test accuracy was chosen to be reported. The cross-entropy was employed as the selection criterion at each node. Moreover, for the bagged decision tree algorithm the number of estimators was selected from [6, 20] with step 2. The ensemble technique (bagging) was applied in order to reduce an error of DT, as the combination of several weak predictors into one high-quality ensemble model improves predictive performance [66]. For the SVM model, different kernel functions (linear, polynomial, radial base function, and sigmoid) were tested. In the case of the polynomial kernel, the degree of the polynomial was selected from [2, 6]. To train each model, fivefold cross-validation was employed and the average test accuracies along with the standard deviation of the accuracies was reported. Classifiers were trained using Python Scikit-learn [67] library.

The computations were performed using MATLAB R2019b Classification Learner App run on a PC.

#### Artificial Neural network with Bayesian regularization algorithm for sorted and unsorted data

ANN:BR has the advantage of tuning the incoming patient data sets. The term epoch in ANN is defined as the measure of the number of times all of the training vectors were used to update the weights [68]. The softmax activation function was used to introduce non-linearity into the model. The inputs were turned into a linear model (ωx + b), where ωx is the matrix multiplication of weights (ω) and inputs (x) and b is the bias. The scores obtained from this step were fed into the softmax function (2) which converts them into probabilities.

2 $$\sigma \left( z \right)_{j} = \frac{{e^{{z_{j} }} }}{{\mathop \sum \nolimits_{k = 1}^{K} e^{{z_{k} }} }} for j = 1, 2 \ldots , k$$

Softmax function maps the set of outputs onto inputs. In this case, there are five outputs which when passed through the softmax function get distributed according to probability (0,1). This was useful for finding the most probable occurrence or classification for a particular output.

A Bayesian regularized neural network (ANN:BR) capable of classifying patients using an assigned HISS score was developed in MATLAB 2018a Neural Network Pattern Recognition App run on a PC [69]. The neural network was trained to a max epoch size of 100 using Bayesian regularization algorithm [70] (training stops according to adaptive weight minimization) using Mean-Square Error as the performance metric. Responses from the five experts were used to create a single label by calculating the mathematical mode as the best metric of central tendency. The mode was chosen over the mean due to possible skew in HISS scores. The NN was trained using (i) sorted and (ii) unsorted data. Sorted data served to ensure that HISS scores were normally distributed among the training and test data. Sorting established groups of 5 different patient data using the 80:20 rule (e.g. 80% of HISS score “1” was used in the training set, while 20% of HISS score “1” was used in the test set). Unsorted data employed no such grouping and hence carried the risk that the test data could be unbalanced in its representation of certain HISS scores. Neural network performance was measured by using a constant test set size of 25 with 4 or fivefold cross-validation, where the training set size varied from 15 to 75% of the total data set size. In yet a totally different and additional approach, the mean test and mean training accuracy were determined by varying the size of the training set between 30 and 80 instances with steps of 5. To test the trained models, a fixed set of instances of size 20 was used. The experiment for each training set size was repeated twenty times to reduce the effect of variance on the results and the mean accuracy was reported. This served as a self-consistent approach across all classifier algorithms.

#### Possibility rule-based classifier

In the possibility rule-based classifier system using function approximation (PRBF) [71], possibility theory is used to handle uncertainty in expert knowledge. The degree of belonging of an instance to the $k$th class may be characterized by $u^{k} \in \left[ {0,1} \right].$ Different theoretical frameworks have been proposed to solve problems that suffer from uncertainty [72] including probability theory, set theoretic functions, and possibility theory. Under the possibility theory [71, 73] framework, $u^{k}$ is the level of possibility that the given data point belongs to the class of score k and the following representation holds for the set of possibilistic classes assigned to the $i$th instance:

3 $$\varvec{u}_{i} = \left( {u_{i}^{1} , u_{i}^{2} , \ldots ,u_{i}^{c} } \right) \forall u_{i}^{k} \in \left[ {0,1} \right]$$

In (3), c is the number of scores defined for the problem, i.e. in this case five scores, being LOW (0), GUARDED (1), ELEVATED (2), HIGH (3), SEVERE (4). Unlike the probabilistic labels, the values of the vector $\varvec{u}_{i }$ do not have to sum up to unity. Instead, each parameter takes a value ranging from 0 to 1. The classification scheme proposed by Nazmi and Homaifar [71], namely, possibility rule-based classifier using function approximation (PRBF), employs this definition of a class assignment and trains a rule-based evolutionary model that given a data point, predicts the degree of possibility to which the SFRP data set belongs to each of the possible classes.

The possibility rule-based classifier was implemented using Python 3.7.5 run on a PC. A fivefold cross-validation was used with population size = 4000, stretch = 25, learning rate = 0.1, and training iterations = 100,000. Having the assigned scores from five expert physicians for the generated SFRP data sets, it is probable that any two physicians might disagree on the score of any one patient’s values or the same physician assigns different scores to patient’s values that are nearly similar. This problem may be addressed with the use of possibility theory [71], capturing the inherent intra-expert and inter-expert variation in the responses of physicians. More specifically, scores provided by the physicians for each set of measurements, the SFRP data set, were converted into possibility values that were values between 0 and 1. For a given measurement vector ${\mathbf{x}}$ and a hypothetical class, $\omega^{k}$, the possibility distribution, $\pi_{{\mathbf{x}}}$, defined for ${\mathbf{x}}$ represents the knowledge contribution of an information source about the actual state of ${\mathbf{x}}$. In other words, $\pi_{{\mathbf{x}}} \left( \omega \right) = 0$ means that state $\omega$ is rejected as impossible, and $\pi_{{\mathbf{x}}} \left( \omega \right) = 1$ means that state $\omega$ is totally possible (plausible). In a machine learning framework, this concept is employed to solve classification problems by taking $\pi_{{\mathbf{x}}}$ to represent the degree of belonging of SFRP data to classes which are provided by the expert(s) [60].

PRBF has two main mechanisms to generate a problem solution; a rule-based evolutionary algorithm to approximate possibility labels, and an information fusion method to make plausible inferences for unseen data. When trained on a dataset with possibility labels, PRBF iteratively evolves a population of overlapping rules which are piece-wise linear approximations of the target possibility distributions. Moreover, the data fusion technique employed in PRBF combines the data provided by multiple sources, i.e., rules of the model, and calculates the most plausible values for the class membership of the unseen data set. Consequently, for an unseen patient data set, the model generates a possibility distribution (π). This distribution may then either be interpreted by an expert for decision-making purposes or processed to extract a crisp class by taking the one with the highest possibility. To demonstrate the benefit of employing a model that is robust in the presence of HISS score uncertainty, the same training data that were generated using SFRP data generator were used to train the PRBF algorithm and the trained model was evaluated against the 100 instances used in the previous sections for the model evaluation. The disagreement among the physicians’ evaluations, was captured by repeating the process for all of the 100 SFRP samples by calculating a set of possibility labels as well as a class label based on the majority vote.

### Performance metric, cross-validation, adequacy of patient data size and predicted patient data size with the number of experts

In general, the performance of a multi-class classification can be measured using accuracy, precision and F-score [74]. A confusion matrix plot can be used to evaluate the quality of the classifier [75]. The matrix contains values corresponding to true labels and predicted labels. The values in the major diagonal of the confusion matrix can determine how well the classifier has performed. In this work accuracy was used as a performance metric to report the prediction performances, which can be obtained from the major diagonal elements of a confusion matrix as follows in (4),

4 $$Accuracy = \frac{\#\,Correct\,predictions}{\#\,predictions} = \frac{{\varSigma }\,of\,elements\,in\,the\,major\,diagonal}{\#\,of\,elements}$$

Cross-validation [76, 77] helps with using all the available data for model training and hence in making more robust predictions. To do so, the data were randomly split into equal sets for training of multiple models. Here a fivefold cross-validation [78] was used. The adequacy for the patient data size was tested with the minimal point for stabilizing validation accuracy. The adequacy for the number of experts and the prediction for the patient data size for a test accuracy of 0.99 and 0.999 with the predicted number of experts necessary to achieve that accuracy was arrived at using the regression model fit and application of predictive modeling in JMP Pro software version 14.0 run on a PC.

### Comparison of classification algorithms

The classification algorithms employed in the previous sections were compared for their respective accuracies. In this case, DT, SVM and PRBF classifiers were trained according to the approach presented in “Multi-class linear support vector machine and ensemble bagged decision tree classifiers” and “Possibility rule-based classifier” sections, respectively. While for ANN:BR, the number of nodes was selected from [5, 63] with step 5. Different activation functions were tested and a ‘tanh’ function was selected. The solver that was used to train the models was the ‘adam’ solver and the model was trained for 100,000 iterations. Finally, for the PRBF model, the maximum number of rules was selected from {500, 1000, 3000, 4000, 5000, 6000}. The maximum condition stretch was selected from [20, 27, 32, 34, 38] which modifies the proportional size of the rule condition and effects the accuracy of the rules. The learning rate was set to 0.1, and the number of training iterations was 30,000.

To train the PRBF algorithm, the uncertain labels (u) were used and the other classification algorithms were trained on D1–D5 and using the majority vote of the labels obtained from the five physician experts. For the decision tree classifiers, support vector machine and the neural network, their Python implementation that was available in Scikit-learn [67] library was used. For the PRBF algorithm, its implementation in Java was used. All experiments were carried out on a 2.70 GHz Windows 10 machine with a 16.0 GB RAM. One-way Analysis of Variance (ANOVA) was used to determine the significance levels for the performance of these algorithms using JMP Pro software version 14.0 run on a PC.

## Results

### Classification via linear support vector machine and ensemble bagged decision tree

**Fig. 2:**
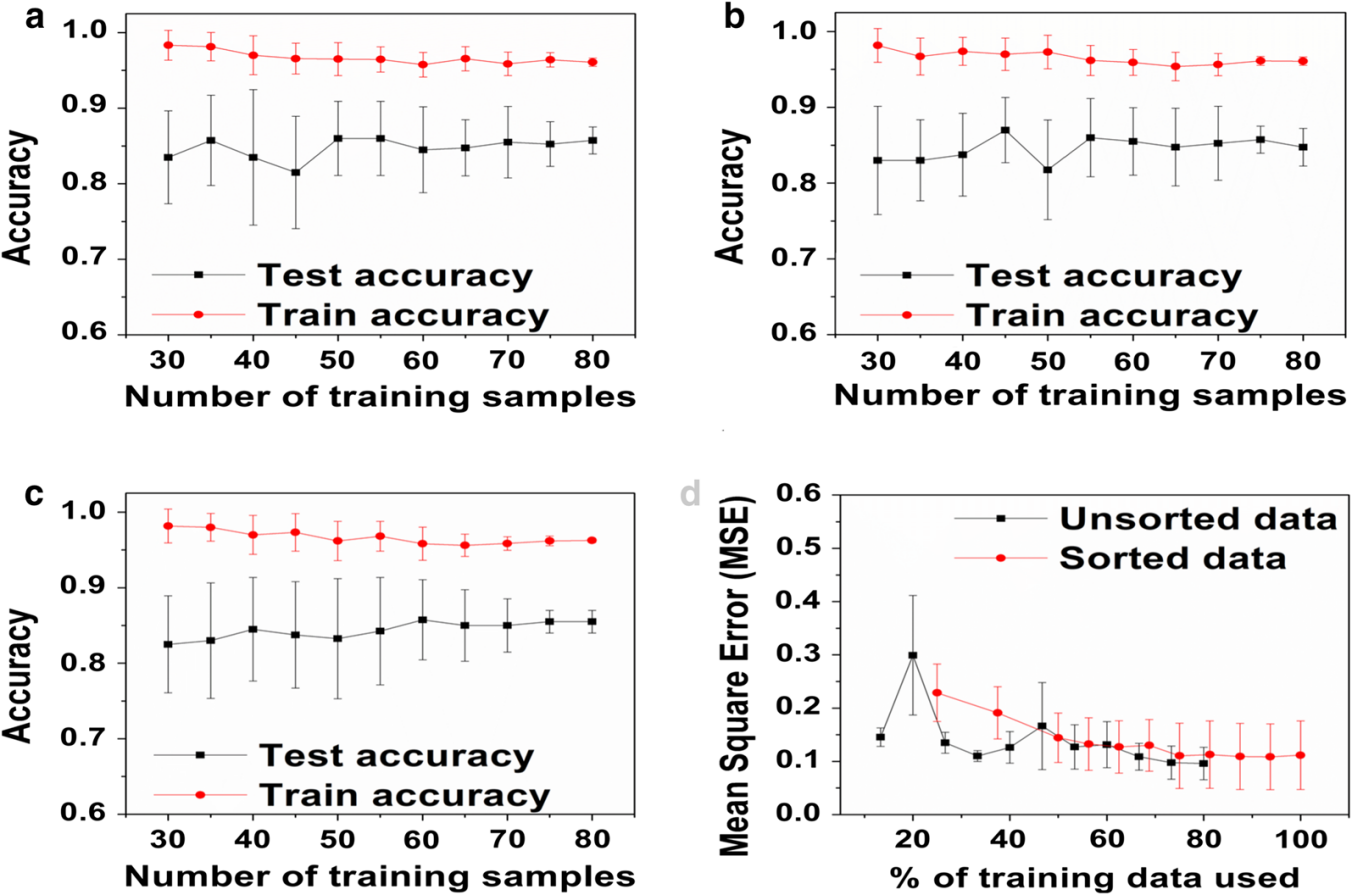
Evaluation of the mean test and mean train accuracy versus the number of training samples for the training size varied from 30-80 in steps of 5. Accuracy vs. training dataset size for **a** SVM-L, **b** EBDT, **c** ANN:BR, and **d** Evaluation of the influence of the size of the training set, expressed as a  % of available data, on the performance of the ANN:BR as expressed in the Mean-Square-Error for maximum epochs of 100. For unsorted data, trained ANNs were tested with a sliding window of 25 validation data set. For sorted data, trained ANNs were tested with a sliding window of 20 validation data set

Two well-established classifier algorithms, namely linear support vector machine (SVM-L) and ensemble bagged decision tree (EBDT), were used in the classification of SFRP data. Figure 2a, b provide the accuracy versus the number of training samples for SVM-L and EBDT. Table 3 presents the findings of the SVM-L and EBDT classifiers in terms of their validation accuracy.

**Table 3:** Application of two different algorithms (linear support vector machine and ensemble bagged decision tree) to the five(5) unique SFRP data sets; [100][D1], [100][D2], [100][D3], [100][D4] and [100][D5]

Class	Frequency (%)				
	D1	D2	D3	D4	D5
0	56	43	37	43	53
1	14	20	27	18	17
2	5	18	7	15	13
3	19	17	11	24	17
4	6	2	18	0	0
SVM-L accuracy (%)	78.3 ± 0.5	92.7 ± 0.5	78.3 ± 2.4	88.3 ± 0.5	86.7 ± 0.9
EBDT accuracy (%)	83.3 ± 1.2	96.3 ± 0.9	72.3 ± 0.9	90.0 ± 0.0	87.7 ± 1.2
Class with the highest confusion (TPR—sensitivity for EBDT)	4 (17%)	4 (0%)	2 (14%)	2 (60%)	2 (77%)

The table presents the fraction of the total observations for each class for each dataset and corresponding cross-validated accuracies for both classifiers. The confusion (true positive rates (TPR)) was correlated to the percentage of observations of the class

Analysis of each experts’ model individually, revealed that EBDT generally performs better than SVM-L (Table 3). Although the differences between both predictors for each dataset were slight (in the range of 2–6%), when it comes to patient stratification decisions, small improvements may be consequential to the therapeutic intervention for a patient. The highest cross-validated accuracy was achieved for the expert D2 dataset and the EBDT classifier (96.3 ± 0.9%). However, a confusion matrix revealed that D2 failed completely to predict Class 4 (Severe), as 100% of labels were misclassified. Among all experts, the highest confusion (TPR) occurred for Class 2 (Elevated), which was the most frequently misclassified as either Class 1 (Guarded) or 3 (High) and for Class 4 (Severe) misclassified as Class 3 (High). There was no single instance where all the five experts concurred on the score of 2. This is due to the fact that 2 is a score in the mid-range of 0–4 and hence higher variability for this score was introduced compared to the extremities [79]. As shown in Table 3, a high level of misclassification may result from an imbalanced number of instances in each class. For example, for expert D1, only 6 instances out of 100 were labeled with Class 4 (Severe), which leads to only 17% TPR. For D3, only two data rows were labeled as Class 4 (Severe), which caused complete misclassification of this score (0% TPR). While for D4 and D5, despite the high performance, none of the input instances were scored as Class 4 (Severe), leading to a model which will fail to make predictions of this class for the new data. Bagging classifiers may reduce the misclassification rate and improve overall accuracy of algorithms. Thus, the EBDT classifier, while being more time-consuming, performed with high accuracy compared to the SVM-L. However, the support vector machine classifiers had a higher accuracy for the data set D3 whereas the decision tree algorithm was less effective in capturing the localized accuracy of D3. From the literature, accuracies of 83–88% for SVM [80], and accuracies of 70–83% have been reported for decision trees in medical applications [81].

### Classification via artificial neural network classifier

With a constant size of 25 validation data sets, it was observed that the error increased with increase of the SFRP training data sets. From the literature, it is known that the error should have stabilized or be shown a decrease to some extent with increasing training data sets [82]. This was attributed to the difference in the opinions of the experts. Consequently, a sliding window of validation data sets was used. Using the approach of Mode to allow the doctors to vote together along with a sliding window of validation data sets showed a decrease in error with an increase in the training data sets, in agreement with the literature as shown in Fig. 2d [83]. Here it was observed that sorting improved output quality with a smooth trend towards equilibrium or limiting error. However, the unsorted data appeared chaotic with stochastic noise.

Unsorted data at a very low training set size (15–20%) showed a very high standard deviation of MSE due to a lack of heterogeneity within the training set. The probability of less frequently available HISS scores, such as 4, being withheld from the training set was very high. For example, at a training set size of 20%, the probability of a HISS Score of 4 showing up in the training set was 0.008. Unsorted data tended to the same MSE as sorted data (~ 0.12), but was variable in its descent due to probabilities of scores not being included in the training set because if low frequency (e.g. HISS Score 4). As shown in Fig. 2d, improvement in the test accuracy of the ANN:BR was insignificant for the number of SFRP training samples larger than 75. Based on Fig. 2c, d, 75 SFRP data sets were established to be an adequate data size to build a model for prediction. From the literature, prediction accuracies of 44% and training accuracies of 50% and above have been reported for neural networks in medical applications [84, 85]. When the accuracies were on a lower side, the neural network approaches were often combined with hybrid fuzzy systems [86].

### Performance of PRBF

One simple way to resolve conflict among class evaluations from multiple experts is to take the class label that was most frequently identified. An alternative approach, which makes better use of the rich data provided by the experts, is to calculate a set of possibility labels using (3), which is expected to reflect the disagreement among experts better than solely taking the majority vote. Table 4A depicts the fivefold training and validation accuracies from experts D1–D4, for the PRBF algorithm. For the sample presented in Table 4B, the majority vote opts for class zero to represent the patient’s status, as shown in Table 4. The possibility labels calculated using the (3) for the same patient data are provided in column ‘Uncertain labels’ in Table 4B. The uncertain labels assume the association of the patient data to class zero and one. The degree of possibility that the sample belongs to each class is different however and is equal to 1 and 0.5 for class zero and one, respectively. This graded association reflects the disagreement among the experts in deciding the true status of the patient.

**Table 4:** Results from PRBF algorithm from experts D1-D4. A) Cross-validation model training results for PRBF algorithm for Population size = 4000, stretch = 25, learning rate = 0.1, and training iterations = 100,000, B) True labels and predicted uncertain labels for the tested SFRP sample of fictitious patient number 72

A		
	Training accuracy	Test accuracy
Fold-1	0.95	0.90
Fold-2	0.96	0.90
Fold-3	0.98	0.95
Fold-4	0.95	0.95
Fold-5	0.94	0.90
Mean accuracy	0.96	0.92
Standard deviation	± 0.01	± 0.03

B			
Fictitious patient	Majority vote	Uncertain label (${\text{u}}$)	PRBF prediction ($\pi$)
72	0	[1,0.5,0,0,0]	[0.979,0.321,0,0,0]

A confusion matrix plot was used to represent the performance of the possibility rule-based classifier using function approximation (PRBF) [75]. Folds indicate the division of the data set to confirm that each of the folds has been used as a set. Uncertainty or error information has been utilized to support medical diagnostics where a prediction accuracy of 87% has been reported for a training accuracy of 90% [87]. Common approaches like possibility rule-based classification for handling error include fuzzy probabilities [88], and hybrid fuzzy-NN systems [89].

The PRBF model was able to predict HISS scores with 92% accuracy for a testing and training accuracy of 96% when using D1–D4. These results confirm that the idea of integrating evaluations from multiple experts and modeling them with a proper uncertainty handling tool, which is possibility theory in this work is beneficial for decision-making. Note that by increasing the number of training samples of the SFRP data sets, the model will be better trained and able to produce more accurate predictions.

### Comparison of the test accuracies of classification algorithms

**Fig. 3:**
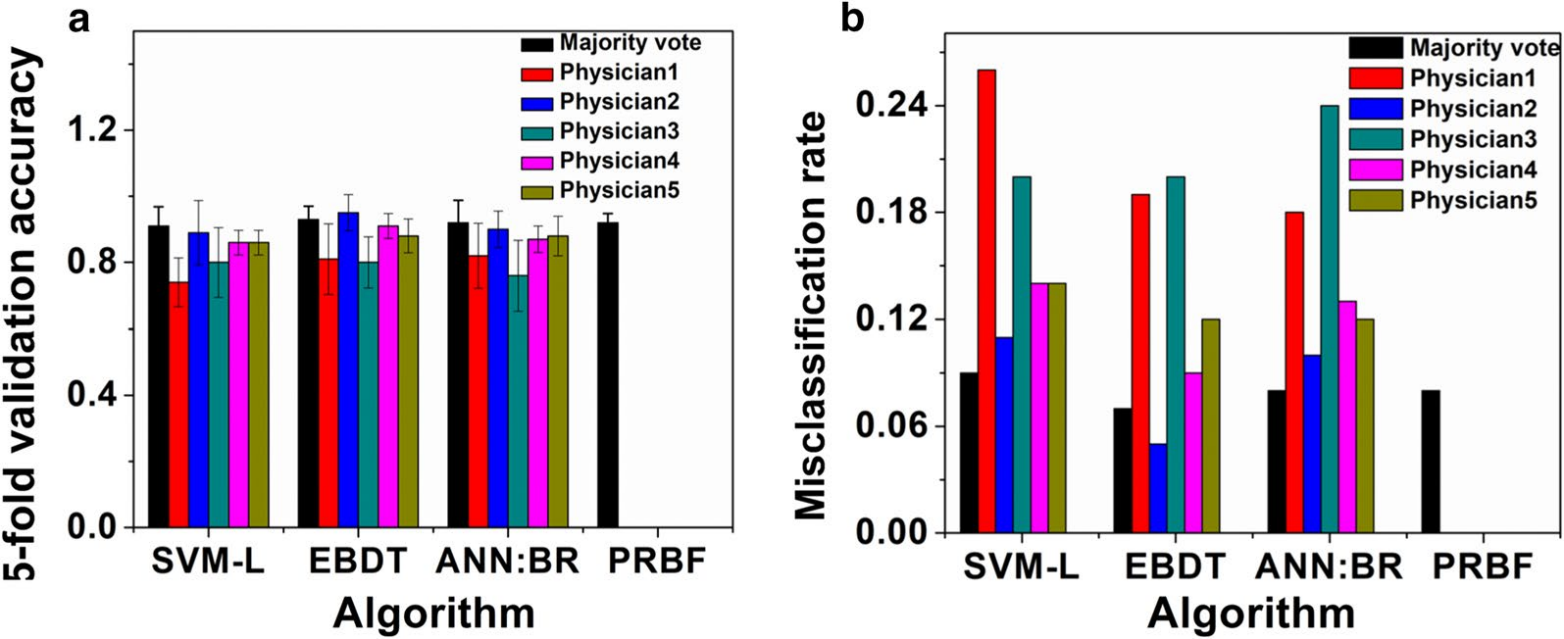
Comparison of **a** Test accuracies, and **b** Misclassification rate, of SVM-L, EBDT, and ANN:BR for experts D1–D5 as well as the majority vote, along with the uncertainty labels of PRBF algorithms for experts D1–D4

The performance of the four classification algorithms, linear support vector machine (SVM-L), ensemble bagged decision tree (EBDT), artificial neural network with Bayesian regularization algorithm (ANN:BR) and possibility rule-based using function approximation (PRBF) were compared for their ability to accurately classify the SFRP data sets. Figure 3a lists the test accuracies and Fig. 3b shows the misclassification rates for the classification algorithms and the uncertainty labels of PRBF algorithm for different experts and the majority vote. The highest accuracy is highlighted in bold for each algorithm. SVM-L, EBDT, ANN:BR and PRBF generated score predictions with testing accuracies (majority vote) corresponding to 0.91 ± 0.06, 0.93 ± 0.04, 0.92 ± 0.07, and 0.92 ± 0.03, respectively, with no statistically significant difference (p > 0.05) in their means for ± 95% confidence interval (C.I).

### Predictions for the adequacy of the patient data size and number of experts for improved accuracy

It is reasonable to ask, given the scoring accuracies obtained for the 100 patients and 5 physician experts 100 [5], what data set size and how many experts will be required to improve scoring accuracies? Targeted accuracies of 99% and 99.9% could be achieved with SFRP data size and clinical expert scores of 147 [7] (99%) and 154 [9] (99.9%), respectively. The model fit for 99% was for a R^2^ = 0.96 with the Total Sum of Squares (SS_total_) as 0.04 with a statistical significance of p ≤ 0.05 for a ± 95% confidence interval (C.I). The model fit for 99.9% was for a R^2^ = 0.89 with the Total Sum of Squares (SS_total_) as 0.11 with a statistical significance of p ≤ 0.05 for a ± 95% confidence interval (C.I).

## Discussion

### Evaluation of individual classifiers

The collection, labeling and archiving of medical data is usually time-consuming, expensive and fraught with security concerns, appropriately so, [77]. Therefore, it is a challenge to build predictive models based on limited available training data. Moreover, the labels are often provided by multiple experts, who may have different opinions about the same patient’s health status. Such disagreement may result from differences in experts’ knowledge and clinical experience. As a worst-case scenario, differences in opinions may lead to patient misclassification, which may have serious consequences to their health [90]. The goal of the present study was to produce and use 100 instances of Sensible Fictitious Rationalized Patient data in the development of predictive models for patient stratification, to use expert opinion to achieve the same stratification in order to ground truth the predictive models and to engage cognizance of intra-expert consistency and inter-expert variability. The study revealed that the more imbalanced the input data, the higher the misclassification penalty. The similarity in misclassification (high level of misclassification of Scores 2 and 4) for each dataset and for both EBDT and SVM-L classifiers, may be the result of insufficient information provided to perform reliable labeling. It is, therefore, extremely important to compare various classifiers in terms of not only their accuracy but also their level of misclassification as performed in this study.

### Qualitative evaluation of experts’ HISS scoring

As a pilot study, the opinions of five experts, D1– D5, were obtained. Expert 1 based his bias weighing decisions on the abnormal levels of biomarkers, being driven by the extremes. For example, when the lactate levels were high, with potassium elevated but compensated, but with a normal pH, this produced a HISS score of 1. It is observed that a score of 2 was assigned when the lactate level does not correlate with other values (normal pH, Eukalemia, Euoxia). High lactate, very low glucose, low pH and normal oxygen produced a HISS score of 3. All values very deranged with pH almost out of physiologic non-recoverable range; hypoxia below 60, elevated lactate, potassium elevated suggesting cell injury, resulted in a HISS score of 4. While providing the scores, expert 2 was able to pick the ones that were similar. Hence, his scores were consistent across all different profiles. The scoring pattern of expert 3 was not localised. Expert 4 localised his scores from 0 to 3. While this paper is not concerned with expert performance, and the data set was far too small to allow the analysis of experts, the very low intra-expert variability (8.0%) and larger inter-expert variability (20.6%) is worthy of mention.

### Comparison of classifiers in terms of cross-validation accuracy

**Fig. 4:**
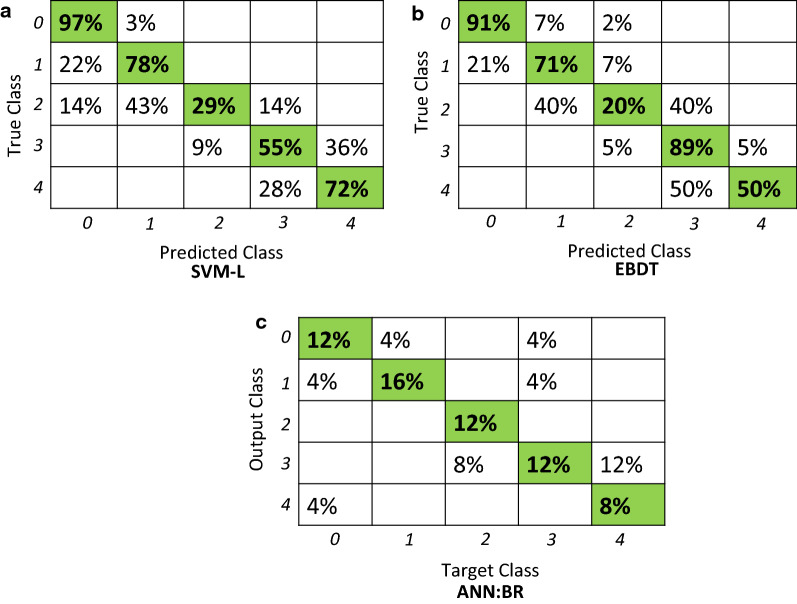
Representative confusion matrices for SVM-L, EBDT, ANN:BR

By the majority vote, SVM-L, EBDT, ANN:BR, and PRBF had cross-validated accuracies of 0.91 ± 0.06, 0.93 ± 0.04, 0.92 ± 0.07, and 0.92 ± 0.03 respectively. The results for SVM-L, EBDT, and ANN:BR were statistically significant. The misclassification is more prominent among the middle classes of 2 and 3. For example from Fig. 4, misclassification rates were 71% for the class of 2 for SVM-L This is because the experts converge upon the extreme values but may have an overlap in the middle classes. There is a need to better delineate the intermediate shock states and so successfully intervene with appropriate resuscitation measures. The accuracy of these intermediate states can be improved by expanding upon the number of expert participants. We hope to improve accuracy of these intermediate states by expanding upon the number of expert participants. Linear support vector machine (SVM-L) and ensemble bagged decision tree (EBDT) classifiers provided for classification in a simple hierarchy of a tree structure and SVM-L provided robust classification. An unquestionable advantage of the presented decision tree classifiers is that they are simple and rapid prediction tools which establishes the trauma severity score with a high accuracy. The results showed that the decision tree classifiers constitute a reasonable basis for the further extensive studies on more specific and complex prediction approaches which may overcome the limitations of the current methods such as a lack of external validation of the model, experts’ opinion, or variation.

#### Performance of PRBF relative to other classifiers

The PRBF classifier added a layer to the intra- and inter- expert variabilities addressed by the other classifiers by tapping into the votes (either majority or individual) of the experts for a particular patient data set and reporting the number of times a physician’s label agrees with the consensus. It was interesting to note that expert 4 had the highest concurrence from his scoring pattern localized for 0–3. This coincides with real-life scenarios when expert physicians try to categorize the patients from 0 to 3 and try to save them. Comparatively, the score of 4 corresponding to severe was rare. From the fivefold cross-validation results, the improvement in the test accuracy is insignificant for the number of training samples larger than 70. The increase in the training samples from 30 to 70% improved the accuracy from 71 to 78.5%.

The PRBF model seeks to incorporate the inherent disagreement among the physician experts into the model training procedure. According to Fig. 3a, integrating evaluations from multiple physicians through the possibility theory resulted in a better performance than SVM-L, EBDT, ANN:BR, and PBRF trained using the majority vote. This implies that employing different tools of modeling the uncertainty, allows for capturing different forms of uncertainty and potentially leads to better prediction accuracy. Moreover, training a model using PRBF allows for an additional level of interpretation of the model prediction during the decision-making process. To illustrate this point, consider the example of Fictitious Patient 72 presented in Table 4B. When the trained PRBF model was elicited for predicting a label for this sample, it was able to correctly predict association to both classes with different degrees of belonging, as shown in Fig. 3a. For each test sample, the PRBF model provides a degree of possibility to belong to each class. The possibility values can be used to gain more insight into the prediction process of the model and provides the decision-maker with more information about the potentially over-lapping classes. Figure 3b shows the misclassification rates for than SVM-L, EBDT, ANN:BR, and PBRF. PRBF has the least misclassification rates. As per the majority vote, SVM-L seems to have high misclassification followed by ANN:BR and then the EBDT. Representative confusion matrices have been shown in Fig. 4.

### Prediction for an adequate patient data size and predicted patient data size with the number of experts

An adequate testing patient data size of 75 was found beyond which the Mean Square Error and the validation accuracy were both stabilized for ANN:BR. This therefore establishes the minimum patient data set needed to conduct predictive patient classification. The present patient data size of 100 and five scoring experts produced accuracies of 0.93. The patient data size needed to obtain an improved accuracy of 0.99 was predicted to be 147 with the predicted number of 7 experts. Similarly, for an accuracy of 0.999, the predicted size of the number of patient data was 154 with 9 scoring experts. From the model, R^2^ was 0.96, with the Total Sum or Squares (SS_total_) as 0.04 with p ≤ 0.05 for a ± 95% confidence interval (C.I). Increasing the number of scoring experts from 5 to 7 can yield an accuracy of 99% but necessitates an increase in patient data set size from 100 to 147 (R^2^ = 0.96). Likewise, increasing the number of scoring experts from 5 to 9 can yield an accuracy of 99.9% but necessitates an increase in patient data set size from 100 to 154 (R^2^ = 0.89). There is less certainty in the prediction in going from 99 to 99.9% because of the limitations of the resent data set.

### Limitations of the current approach and improvements to the existing model-based on a substantial number of experts

The HISS score is not intended to replace existing approaches but rather augment present decision making. This is a preliminary evaluation of the multiple approaches for the fusion of discrete patient sensor data into an actionable HISS score. The interactions among variables for metabolic biomarkers across dataset features and the effects would be captured using machine learning algorithms. Hence, the model-based predictions along with the evaluations of the experts’ opinions form a baseline and serve as a precursor to a larger study for which the following improvement strategies can be implemented:

#### Number of experts

The current study uses five experts, D1-D5, with 100 SFRP data sets. The robustness of the probability theory and capacity to ascertain and account for physician variance was tested by means of uncertainty in the experts’ opinions. From the results, it is observed that the self-consistency in the scoring of 4 experts can overcome the scoring inconsistency of 1 expert. Hence, a ratio of 4:1 is suggested for the number of experts. This aids in substantiating the robustness of the machine learning approaches to ascribe an accurate and actionable HISS score despite the presence of inter-physician variance.

#### Overlap of intermediate shock states

Due to the preliminary small number of experts, there is overlap of the intermediate states. Increasing the number of experts can help to delineate these states and improve accuracy. Future work would entail (1) improving the predictive accuracy of HISS by growing the expert data set and (2) engaging in a small scale preliminary clinical trial to assess feasibility. There is tremendous opportunity to build a longitudinal assessment data set, particularly as it relates to data on extreme resuscitations and long-term patient outcomes.

#### The confidence level of expert scores

It is believed that experts assign the patients to a particular class with a certain confidence level, in this case, 100%. However, they can be requested to reveal their confidence level in scoring each patient. Alternatively, the statistical confidence can be extracted by capturing the variability in the responses of the physicians using approaches like ANOVA. This could be implemented for a substantial number of experts (e.g. 100).

#### The relative weights of each patient attribute

In arriving at the class assignment, the expert physician reviews the five relevant physiological attributes. In its implementation, the classifier algorithms accept a single score with the assumption that each attribute is equally weighted in that decision-making assignment by the expert. In reality, experts inherently weigh each attribute and the weight is often influenced by that value and the values of other attributes. Based on their experience, the expert physician may treat certain biomarker attributes as being more or less important/influential than others when assigning the patient to the selected class. This can be extended for a substantial number of experts (e.g. 100), where a methodology can be developed to extract the relative weighting of each attribute. This relative weighting is thus a global factor assigned to the attribute. From the multiple expert physician responses obtained, a statistical assessment of the significance of each attribute can be determined. Techniques such as “leave one out analysis” and ANOVA will allow the extraction of the relative sensitivity of each attribute to the class assignment.

#### Temporal variation in HISS scores

In the present implementation, SFRP data were presented to each classifier algorithm as STAT data. However, patients are known to display temporal changes or trends in these biomarker values during hemorrhage progression such as during evacuation from theatre to the Green Zone. There is increasing attention being given to the diagnostic relevance of trend data in patient stratification. Temporal variations in the data to reveal physiological trends can be explored using algorithms like recurrent neural network; however, this is outside the scope of the present study.

#### Clinical significance and integration of HISS

HISS is proposed for use as an adjunct device in conjuction with the state-of-the-art emergency protocols. It would help trauma surgeons and emergency physicians in mass triage and point of care stabilization of traumatic hemorrhage patients. Presently used prediction models like the TRISS or RISC-2 have been useful, being aggregates of gross physiological trends. HISS would serve as a supplement to current clinical algorithms as these can be improved upon especially by developing approaches that are capable of measuring and indicating real-time high resolution time series data that can be used for real-time decision making. Furthermore, this study may expand upon biological interactions and the relative contributions of each clinical/physiological parameter such as to inform decision making. This could be applied as a precision health approach to the diagnosis and treatment of victims of traumatic shock.

## Conclusions

In this study, the Sensible Fictitious Rationalized Patient (SFRP) synthetic data generator was introduced for hemorrhaging trauma patients wherein five biomarkers; glucose, lactate, pH, potassium, and oxygen tension, served as the basis for an actionable HISS score rendered by five experts. This score is intended to serve as an adjunct and be complementary to current measures. The focus is on metabolic biomarkers over the traditional gross physiological data. Normalization of these values may greatly assist in preventing the under and over-resuscitation of victims. Several classification algorithms; linear support vector machine (SVM-L), ensemble bagged decision tree (EBDT), artificial neural network with bayesian Regularization algorithm (ANN:BR) and possibility rule-based using function approximation (PRBF) were evaluated for their ability to accurately classify the 100 entries of the SFRP data set. These data-driven predictions are presented as an adjunct to help the decision-making of physicians regarding the status of the hemorrhaging patient during triage and uses a severity scale of (0 = LOW, 1 = GUARDED, 2 = ELEVATED, 3 = HIGH, 4 = SEVERE). A training data set size of 75 has been identified as adequate to achieve the best performance by minimizing the Mean Square Error. This approach has the advantage of high validation accuracies from the ensemble bagged decision trees and linear support vector machines (93 ± 0.04% and 91 ± 0.06%) with the tunability of neural networks (92 ± 0.07%), and the ability to capture the uncertainty in the responses of experts with the help of a possibility theory-based approach (92 ± 0.03%). The predictions generated using the classification methods would assist in an adjunct device in the form of a biosensor system for point-of-care monitoring of the trauma patient, especially in mass casualty situations.

Improvement strategies are discussed with an increase in the number of experts to 100 scoring the SFRP data sets. This paper has a clinical utility in terms of classification by grouping data, prediction for incoming data and regression by means of prediction of continuous data. The predicted patient data size to obtain a test accuracy of 0.99 has been identified to be 147 with a predicted number of 7 experts. Refined prediction model disclosed a predicted patient data size of 154 with a predicted number of 9 experts for a test accuracy of 0.999. Similarly, the adequacy of the patient data size has been identified to be 75 and of the number of experts has been noted as 5 to allow training and validation. Intermediate states reveal more overlap when compared to the extreme states of LOW and SEVERE. HISS may be clinically relevant as it relates to the translation of physiologic states to severity and outcomes. Future work will entail improving the predictive accuracy of HISS and delineating the intermediate shock states by growing our expert data set and engaging in a small scale preliminary clinical trial to assess feasibility.

## Data Availability

All data used in the manuscript will be provided upon request to the corresponding author.

## Abbreviations

HISS: Hemorrhage Intensive Severity and Survivability score
SFRP: Sensible Fictitious Rationalized Patient data
SVM-L: Linear support vector machine
EBDT: Ensemble Bagged Decision tree
ANN:BR: Artificial Neural Network with Bayesian Regularization
PRBF: Possibility rule-based using function approximation
MODS: Multiple Organ Dysfunction Syndrome
START: Simple Triage and Rapid Treatment
SAVE: Secondary Assessment of Victim Endpoint
ICU: Intensive care unit
ISS: Injury severity score
AIS: Abbreviated Injury Scale
PSM: Physiologic Status Monitoring
DeepSOFA: Deep Sequential Organ Failure Assessment
HIPAA: Health Insurance Portability and Accountability Act
ANOVA: One-way analysis of variance
TPR: True positive rates
MSE: Mean square error
NN: Neural network
C.I: Confidence interval
SS_total_: Sum of squares
